# West Nile Virus Lineage 2 Spreads Westwards in Europe and Overwinters in North-Eastern Spain (2017–2020)

**DOI:** 10.3390/v14030569

**Published:** 2022-03-09

**Authors:** Pilar Aguilera-Sepúlveda, Sebastián Napp, Francisco Llorente, Carlos Solano-Manrique, Rafael Molina-López, Elena Obón, Alba Solé, Miguel Ángel Jiménez-Clavero, Jovita Fernández-Pinero, Núria Busquets

**Affiliations:** 1 Centro de Investigación en Sanidad Animal (CISA), Instituto Nacional de Investigación y Tecnología Agraria y Alimentaria (INIA-CSIC), 28130 Valdeolmos, Spain; aguilera.pilar@inia.es (P.A.-S.); dgracia@inia.es (F.L.); majimenez@inia.es (M.Á.J.-C.); 2 Unitat Mixta d’Investigació IRTA-UAB en Sanitat Animal, Centre de Recerca en Sanitat Animal (CReSA), Campus de la Universitat Autònoma de Barcelona (UAB), Bellaterra, 08193 Barcelona, Spain; sebastian.napp@irta.cat; 3 IRTA, Programa de Sanitat Animal, Centre de Recerca en Sanitat Animal (CReSA), Campus de la Universitat Autònoma de Barcelona (UAB), Bellaterra, 08193 Barcelona, Spain; 4 Centre de Fauna de Vallcalent, Àrea de Gestió Ambiental Servei de Fauna i Flora, Forestal Catalana, 25199 Lleida, Spain; carlos.solano@gencat.cat; 5 Centre de Fauna de Torreferrussa, Àrea de Gestió Ambiental Servei de Fauna i Flora, Forestal Catalana, 08130 Santa Perpètua de Mogoda, Spain; rafael.molina@gencat.cat (R.M.-L.); elena.obon@gencat.cat (E.O.); 6 Departament d’Acció Climàtica, Alimentació i Agenda Rural, 08007 Barcelona, Spain; asoles@gencat.cat; 7 CIBER of Epidemiology and Public Health (CIBERESP), 28029 Madrid, Spain

**Keywords:** West Nile virus, lineage-2, Spain, phylogeny, avian host, northern goshawk, overwintering, Europe

## Abstract

West Nile virus lineage 2 (WNV-L2) emerged in Europe in 2004; since then, it has spread across the continent, causing outbreaks in humans and animals. During 2017 and 2020, WNV-L2 was detected and isolated from four northern goshawks in two provinces of Catalonia (north-eastern Spain). In order to characterise the first Spanish WNV-L2 isolates and elucidate the potential overwintering of the virus in this Mediterranean region, complete genome sequencing, phylogenetic analyses, and a study of phenotypic characterisation were performed. Our results showed that these Spanish isolates belonged to the central-southern WNV-L2 clade. In more detail, they were related to the Lombardy cluster that emerged in Italy in 2013 and has been able to spread westwards, causing outbreaks in France (2018) and Spain (2017 and 2020). Phenotypic characterisation performed in vitro showed that these isolates presented characteristics corresponding to strains of moderate to high virulence. All these findings evidence that these WNV-L2 strains have been able to circulate and overwinter in the region, and are pathogenic, at least in northern goshawks, which seem to be very susceptible to WNV infection and may be good indicators of WNV-L2 circulation. Due to the increasing number of human and animal cases in Europe in the last years, this zoonotic flavivirus should be kept under extensive surveillance, following a One-Health approach.

## 1. Introduction

West Nile virus (WNV) (genus *Flavivirus*, family *Flaviviridae*) is one of the most widespread zoonotic arboviruses in the world, as it is present in Africa, Europe, the Middle East, Asia, and the New World [1]. It circulates in an enzootic cycle where mosquitoes (mainly *Culex* spp.) act as vectors and birds are the reservoir hosts. Many bird species can act as competent hosts [2]. Humans and horses can also be infected and may develop a neurologic disease; however, they are considered dead-end hosts, as the viraemia resulting from the infection is insufficient to infect a new vector. In humans, WNV has also been transmitted in a very limited number of cases by transfusions of blood, organ transplants, and during pregnancy, delivery, or breast-feeding. Over 80% of WNV infections in humans are asymptomatic and the most prevalent clinical manifestation is West Nile fever (WNF) [3]. However, severe neuroinvasive illness can occur mainly in elderly and immunocompromised patients and about 1 out of 10 people who develop severe illness affecting the central nervous system die. There is not specific prophylaxis or treatment for humans, unlike horses, in which the disease can be prevented with some commercial vaccines [4]. In horses, WNV may cause encephalitis or encephalomyelitis that may provoke clinical signs such as ataxia, incoordination, weakness, muscle fasciculation, and cranial nerve deficits. In addition to the consequences on public health, the impact of WNV on animal health may be significant since the equine sector represents a valuable socio-economic resource in rural areas in Europe, and especially in Spain.

The WNV genome consists of a single-stranded positive-sense RNA genome of approximately 11,000 nt, which encodes and translates a single polyprotein of 3434 amino acids. This polyprotein is further processed by viral and host proteases to yield three structural proteins, –capsid (C), membrane (prM/M), and envelope (E)-, and seven non-structural (NS) proteins (NS1, NS2A, NS2B, NS3, NS4A, NS4B, and NS5), all of them playing an essential role in the viral genome replication [5].

Currently, there are at least eight known lineages of WNV [6], showing its high genetic diversity. Of them, lineages 1 and 2 are the most widely distributed and are responsible for the largest outbreaks in humans and animals.

West Nile virus linage 1 (WNV-L1) was first reported in Europe in 1962, concretely in the Camargue [7]. After that, it caused sporadic outbreaks in the following decades in different European countries. Nevertheless, it started to be considered as a pathogen of concern in this continent after the 1990s, and especially since the 2000s, when it re-emerged. Since then, human and animal cases have multiplied across Europe [8].

West Nile virus lineage 2 (WNV-L2) was first detected in Europe in 2004, in a northern goshawk (*Accipiter gentilis*) from Hungary, being the first report of this lineage outside Africa [9]. This strain (belonging to the defined central-southern European clade) spread rapidly and caused important outbreaks in Hungary and Austria between 2008 and 2009 [10], in Greece in 2010 [11], in Serbia in 2012 [12], and in Italy in 2013 [13]. At the same time, a different WNV-L2, belonging to the named Russian/Romanian clade, affected humans in Russia (2004–2007) [14] and Romania in 2010 [15].

More recently, WNV-L2 has emerged in France [16], Germany [17], the Czech Republic [18], and the Netherlands [19], causing outbreaks in wild birds, and incidental cases in horses and humans. Furthermore, the situation in Italy deserves special attention, as WNV-L1 strains of the western Mediterranean subtype predominated in the region until 2011–2013, when WNV-L2 emerged for the first time in co-circulation with WNV-L1. Since then, lineage 2 has been the most frequently identified, even replacing lineage 1 across the country and originating important outbreaks in humans [13,20,21].

Frequently, after a first outbreak is reported, the disease re-occurs in the same area in the following years, indicating the success of WNV in settling down under favourable conditions.

Regarding the situation in Spain, WNV-L1 has been present in the south and central regions of the country during the last two decades [3,22]. Indeed, it has been responsible for the greatest human outbreak in the country during the 2020 transmission season, causing 77 human cases and eight deaths [3]. On the other hand, although WNV-specific antibodies in birds and horses have been detected in Catalonia (north-eastern Spain) during 2010–2019 [23], WNV-L2 was molecularly detected for the first time in 2017 in this area, away from WNV-L1-affected regions of the country. The detection of this lineage in northern goshawks evidenced the capacity of WNV-L2 to spread from central to western regions of Europe [24].

Particularly, Catalonia has implemented a WNV comprehensive surveillance program (including active and passive surveillance in wild birds and horses) and measures were intensified due to WNV-L2 detection in 2017 [24]. In 2020, WNV-L2 was again detected in northern goshawks with clinical signs compatible to West Nile disease. However, there have not been reports of human events due to WNV-L2 in the region yet, contrasting with other European countries where this lineage has been responsible for numerous cases [20,25].

The present study analysed the full-genome sequences, their phylogenetic relationships with other WNV-L2 strains found in Europe, and the phenotypic characteristics of the first four Spanish isolates of WNV-L2, with the aim of characterising these circulating WNV-L2 strains and elucidating the potential overwintering of the virus in this Mediterranean region. These isolates come from the outbreaks that occurred in northern goshawks in Catalonia (north-eastern Spain) during the 2017 and 2020 seasons.

## 2. Materials and Methods

### 2.1. Sample Collection

Within the surveillance program for WNV implemented in Catalonia, birds found dead or that died at the Wildlife Rehabilitation Centres (WRC) had their heads sent to IRTA-CReSA to be diagnosed for WNV in encephalon samples. Between 2017 and 2020, 373 dead birds from 53 different bird species belonging to 23 families were tested (Table S1). The carcasses of the sampled birds were frozen and, once positive results for WNV were obtained, they were further transferred to IRTA-CReSA. Necropsy was performed at BSL-3 facilities and several organs (kidney, liver, heart and lung) were sampled, collected in 500 µL of DMEM with 6% of Penicillin, Dihydrostreptomycin, and Nystatin (PSN) and stored at −75 °C until further laboratory investigation.

### 2.2. Initial WNV Molecular Diagnostics

Tissue samples were mechanically homogenised using polypropylene pestles and viral RNA was extracted using NucleoSpin^®^ RNA Virus (Macherey-Nagel, Düren, Germany) according to the manufacturer’s protocol. RNA was eluted in a final volume of 50 μL of RNase-free water. WNV RNA was detected by real-time reverse transcription PCR (RT-PCR) using the primers and probe previously described [26] and AgPath-ID™ One-Step RT-PCR reagents (Applied Biosystems, Foster City, CA, USA). The amplification was detected using a Real-Time PCR 7500 Fast System (Applied Biosystems) with the following thermal profile: 48 °C for 10 min; 95 °C for 10 min; and then 45 cycles at 95 °C for 15 s and at 60 °C for 60 s. Further analysis of a partial sequence of the NS5 gene, using the primers and RT-PCR conditions previously described by Scaramozzino et al. [27], were performed in the encephalon samples to determine the WNV lineage.

### 2.3. Virus Isolation

WNV isolation from encephalon samples was performed in Vero cells (ATCC, ref. CCL-81) at IRTA-CReSA facilities. Briefy, cells with 90% of confluence were exposed to the supernatant of homogenised samples for 1 h, and then, post-infection media (DMEM with 2% FBS, 2% PSN and 2% Glutamine) was added to incubate the infected cells for 4 days. Then, the supernatant was collected, centrifuged, aliquoted, and stored at −80 °C. Once WNV isolation was confirmed from one aliquot of obtained isolates by using the same RT-PCR described above and by titration (TCID_50_), WNV isolates were sent to CISA-INIA facilities, where they were propagated similarly in Vero cell cultures (second passage).

### 2.4. Full Genome Sequencing

Total RNA was extracted from 100-µL aliquots of infected cell culture supernatants (2nd passage) using Biosprint 15 workstation (QIAGEN, Valencia, CA, USA), according to the manufacturer’s protocol, with a small modification (carrier RNA was added to a final concentration of 5 μg/mL). RNA was eluted in a final volume of 100-μL RNase-free water, aliquoted, and stored at −80 °C until used.

The complete genomes of the isolates were obtained by sequencing 23 overlapping PCR amplicons based on L. Barzon’s previous design (personal communication). From this original design, 12 primers remained unchanged and the rest were newly designed by M. Elizalde and J. Fernández-Pinero (personal communication) or were taken from a previously described protocol [28] (Table S2).

One-step RT-PCR kit (Qiagen) was used to run conventional RT-PCR assays, following the manufacturer’s instructions. Amplified DNAs were purified using ExoSAP-IT kit (GE Healthcare), and then bidirectionally sequenced by automatic dideoxy cycle sequencing techniques, Big Dye Terminator Cycle Sequencing Kit (v3.1), and an ABI 3730 XL DNA Analyzer, using the same primer sets of the RT-PCR assays.

### 2.5. Sequence and Phylogenetic Analyses

Analysis of the sequences and assembly of the full linear genomes were performed by using SeqMan software (DNASTAR, Madison, WI, USA). The genome sequences obtained in this study were submitted to GenBank database under the accession nos. OM037670, OM037671, OM037672, and OM037673. Multiple alignments were performed with the Clustal W program and phylogenetic trees were created using the Maximum likelihood method (both available in MEGA7 software). Bootstrap analyses were inferred from 500 replicates.

### 2.6. Characterisation of the Viral Plaques

For the in vitro characterisation, the four Spanish WNV-L2 isolates (AC564, AC913, AC923, and AC924) were compared with other WNV known and characterised strains (GenBank accession number of the respective complete genome sequences indicated in parentheses): KUN MP502-66, a putative lineage 6 from Malaysia (GU047874); KUN-KJ359-11 lineage 1b from Australia (JX014270); B956, an ancient lineage 2 from Uganda (AY532665); NY-99, a lineage 1 from New York (FJ151394); and SPA07, a lineage 1 isolate from Spain (FJ766331). Thus, these nine isolates were inoculated in Vero cells in 6-well culture plates as previously described [29]. After staining with neutral red, plaques were visually examined at 72 h post-infection and photographed. Plaque sizes were then measured using the ImageJ program and statistical analyses were performed by using Student’s *t*-test.

## 3. Results

Between 2017 and 2020, 5 out of 373 wild birds found dead or that died at the WRC tested positive by RT-PCR for WNV in encephalon samples. Further analysis of a partial sequence of the NS5 gene indicated that all the positive samples belonged to WNV-L2. All these WNV-L2 infected birds were northern goshawks with clinical signs compatible with WNV infection and were transferred to WRC, where they died or were euthanised.

**Figure 1:**
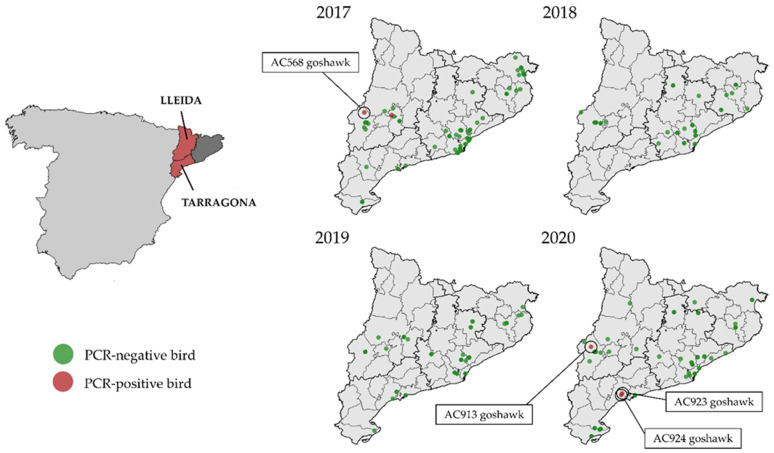
On the left: Provinces were the study was carried out (dark grey and red). In red: location of the affected provinces of Catalonia: Lleida, and Tarragona, within Spain. On the right: distribution of the RT-PCR-positive and negative wild birds sampled in Catalonia between 2017 and 2020.

In detail, in September 2017, a northern goshawk was found with dehydration, apathy, and low weight near an urban area, a municipality called Alguaire, located 15 km northern of the city of Lleida (Catalonia, Spain), and was transferred to the WRC of Vallcalent. Five days later it developed nervous symptoms (head-shaking, incoordination, and inability to stand upright), and was euthanised. This specimen was further codified as AC568 goshawk (Figure 1). Within days, another sick northern goshawk was admitted to the same WRC and died after 2 days [24].

In September 2020, a sick juvenile northern goshawk was collected in the municipality of Alpicat, located 6 km northwest of Lleida (Catalonia, Spain), and was transferred to the WRC of Vallcalent. It was found with a low weight (508 g). Moreover, it showed blepharitis in the left eye and had a corneal ulcer, possibly due to a contusion. Four days later it was euthanised because it showed apathy, incoordination, and an inability to stand upright. This specimen was further codified as AC913 goshawk. At the same time, in September 2020, two sick juveniles of northern goshawks were found in the Tarragona province (Catalonia, Spain) and were transferred to WRC of Torreferrussa. The specimens presented mild to moderate obtundation and were unable to fly. Muscle tone was normal to mildly increased. They had a right head tilt and intermittent cervical dystonia with the head and neck turned to the right. Severe ambulatory tetraparesis and ataxia affecting the wings and the pelvic limbs were observed. Menace response was bilaterally absent. Episodes of quick dilation and constriction of their pupils (eye pinning) were also detected, most likely representing a strong stress or excitement. Thus, they were euthanised. These specimens were then codified as AC923 and AC924 (Figure 1).

Virus isolation from encephalon samples could be carried out in four (AC568, AC913, AC923, and AC924) out of five WNV-positive northern goshawks. No macroscopic lesions were in the northern goshawks affected apart from hematoma that was observed in the brain of AC568, which probably was as consequence of a traumatism due to head-shaking.

WNV real-time RT-PCR analysis showed positive results in all samples examined (Ct values between 14.47 and 35.91) except for the liver, heart, and lung of the AC568 goshawk that were negative (Table 1).

Four full genome linear sequences of 10,972, 10,957, 10,975, and 10,973 nt were obtained from the isolates named AC568, AC913, AC923, and AC924, respectively.

Phylogenetic relationships between the new isolates and other WNV-L2 isolates with full genome sequences available in GenBank were established. Firstly, these four isolates were compared with 45 isolates of WNV-L2, including recent European ones from both main defined clades [30] and representative African sequences.

**Figure 2:**
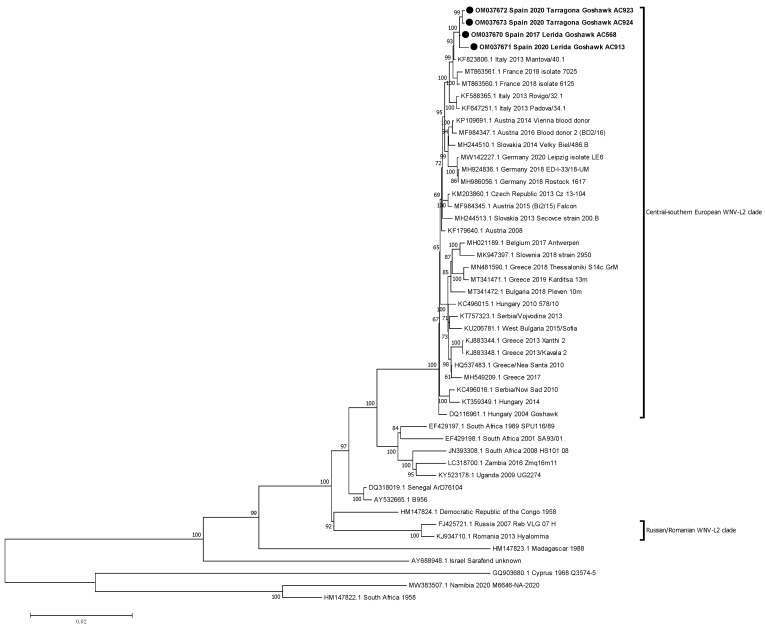
Phylogenetic analysis of complete genome nucleotide sequences of WNV-L2. The evolutionary distances were computed using the optimal GTR+I model, and the phylogenetic tree was constructed with the Maximum likelihood method. Bootstrap values are given for 500 replicates. Viral sequences are identified by GenBank accession number, country, and year of isolation. Sequences emphasised in bold and with a black circle (●) were generated during this study.

**Figure 3:**
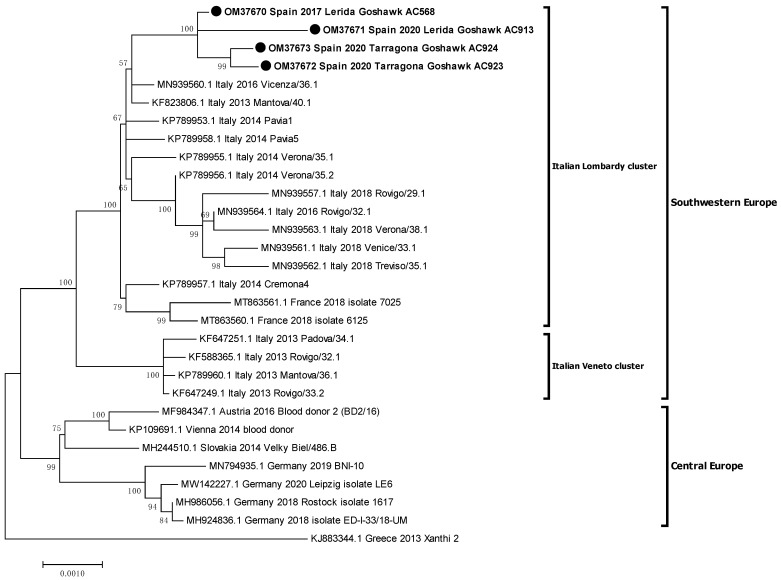
Phylogenetic analysis of complete genome nucleotide sequences of recent WNV-L2 isolates from western-central Europe. Italian clusters were defined by Barzon et al. 2015 [31]. The evolutionary distances were computed using the optimal TN93+G model and the phylogenetic tree was constructed with the Maximum likelihood method. Bootstrap values of major branches are given for 500 replicates. Viral sequences are identified by GenBank accession number, country, and year of isolation. Sequences emphasised in bold and with a black circle (●) were generated during this study.

Phylogenetic analysis placed the Spanish isolates within the central-southern European WNV lineage 2 clade, also defined as the Hungarian clade. Moreover, Spanish isolates were closely related with the Italian (2013) and the French (2018) ones, creating a monophyletic group and slightly differing from the Austrian-German group, which comprises representatives from 2014 to 2020 (Figure 2). Then, a further phylogenetic analysis was performed to refine the closest relationships. The Spanish representatives were analysed with 26 selected sequences of the central-southern European clade, incorporating 14 additional sequences, mostly from Italy. The Spanish isolates specifically grouped together with the Italian Lombardy cluster, according to Barzon et al.’s 2015 definition [31], which also incorporates the French isolates (Figure 3).

The comparison of the Spanish 2017 isolate with the 2020 ones revealed a nucleotide divergence from 0.09% to 0.27%, being isolates AC923 and AC924 (both from the Tarragona province, 2020) the most closely related (presenting a 99.91% of nucleotide homology), and the AC913 isolate (from Lleida 2020) the most dissimilar (99.73% nucleotide homology).

A study of the viral polyproteins was performed comparing Spanish WNV-L2 isolates with representatives of the Lombardy (Italy 2013 KF823806 and France 2018 MT863560) and Veneto (Italy 2013 KF647249) clusters; Hungary 2004 isolate (DQ116961) was used as reference. This study revealed a range of amino acidic divergence from 0.03% (between AC913 and AC568) to 0.41% (between the Hungarian isolate and AC923 and AC924). Comparing the Spanish strains, the variation was 0.03–0.12% (Table S3).

Remarkably, WNV-L2 isolates detected in Spain were characterised by three unique amino acids changes (i.e., NS1-I123F, NS1-H293R, and NS5-T277I) that were absent in the representatives of the Lombardy and Veneto clusters. Additionally, Spanish isolates from 2020 presented exclusive amino acids changes: AC913 showed one single change: NS1-G41S; AC923 presented two single changes: C-M34V and NS1-M113I, and a change shared with AC924: NS4B-N11S; finally, AC924 showed two unique changes: prM-A29V and NS5-R599I. Particularly, most of the variation in the amino acid sequences compared in this study was concentrated in the NS1 protein (Table 2).

**Figure 4:**
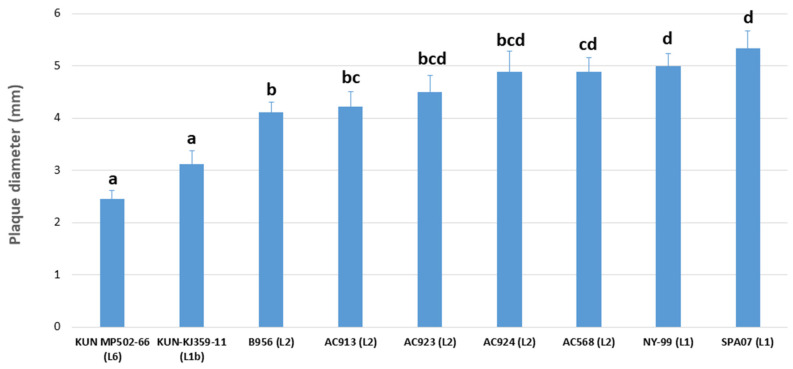
Analysis of the plaque size of WNV isolates. Diameter of the plaques is expressed in millimetres. Name of the strains and lineages are indicated. The letters a, b, c and d indicate statistically significant differences at *p* < 0.05. Columns with no common letters are statistically different (*p* < 0.05).

Capacity of cell infection, as a rough approach to virulence estimation of the isolates, was measured by plaque size assays using data from previous studies [6,32,33]. Based on previous observations [6], Malaysian (L6) and Kunjin isolates (L1b) were considered of low virulence, B956 (L2) was of moderate virulence, and NY-99 and SPA07 (both L1) were classified as isolates of high virulence. As expected, a correlation between virulence and plaque size could be observed in previously known isolates examined in this study. Low virulent strains (i.e., KUN MP502-66 and KUN-KJ359-11) formed small plaques in Vero cells cultures (mean: 2.775 mm), high virulent (i.e., NY99 and SP’07) showed big-size plaques (mean: 5.073 mm), and moderate virulent isolates (i.e., B956) formed plaques of medium size (mean: 4.165 mm) (*p* ≤ 0.05). Considering these categories, Spanish AC568 showed the largest plaque size among all WNV-L2 Spanish isolates examined in this study (mean: 4.89 ± 0.78 mm). This size was statistically larger than the medium-size group (moderate virulence) and most similar to the highly virulent isolates NY-99 and SPA07. On the other hand, AC913 plaques (mean: 4.22 ± 0.83 mm) presented non-significant differences with medium plaque size (B956, moderate virulence), but were statistically distinguishable from the big-size (NY-99 and SPA07) and the small-plaque size (KUN MP502-66 and KUN-KJ359-11) representatives. Finally, AC923 and AC924 plaques (mean: 4.50 ± 0.94 mm and mean: 4.89 ± 01.17 mm, respectively) showed an intermediate phenotype, between isolates with moderate (medium plaque size) and high virulence (big plaque size) (Figure 4).

## 4. Discussion

WNV-L2 was firstly detected in Europe in Hungary in 2004 [9]; since then, it has spread through the continent, reaching Greece (south-eastern) in 2010 [11], Spain (south-western) in 2017 [24], and Germany (northern Europe) in 2018 [17]. This apparently fast speed of spread, compared with the European WNV-L1, contrasts the North American scenario, where WNV-L1 was able to reach the west coast only 4 years after its emergence in New York in 1999 [32]. This fact reveals strong differences between both continents regarding vector-hosts biodiversity, WNV ability to spread, susceptibility, and immunological status of individuals, etc.

The present study focused on the first detections of WNV-L2 in the Iberian Peninsula. The first cases appeared in 2017 in northern goshawks from Catalonia (north-eastern Spain) [24]. Seroconversion was observed in 14 bearded vultures (*Gypaetus barbatus*), a near threatened-species, at the same time in the same WRC [24]. WNV-L2 was not detected again until 2020, when three more northern goshawks were affected in the same (Lleida) and in an adjacent province (Tarragona). WNV-L2 was isolated from encephalon samples, which showed the highest viral loads indicating that this tissue is suitable for diagnosis and viral isolation from northern goshawks infected with WNV-L2. Moreover, neurological clinical signs, which all the affected northern goshawks presented, correlate perfectly with these high viral loads found in the central nervous system. Even though only five RT-PCR positive birds have been detected in Catalonia, recent studies carried out in the region [23] indicated that WNV circulation, not exclusively in birds but also in horses, is much more widespread, although the majority of infections are subclinical.

In 2017, the first isolate of WNV-L2 in Spain was partially sequenced, specifically, a segment of 930 nt of the NS5 gene [24]. In the present study, the full-genome of that isolate and three more recent ones (2020) have been analysed. More in detail, these four isolates showed a degree of nucleotide homology ranging between 99.73–99.91% and a range of amino acid homology between 99.88–99.97%, sharing three exclusive amino acid positions. These data evidence that this strain has remained genetically stable since its first detection in 2017 in Lleida and has managed to overwinter in the area with some geographic spread to another neighbouring province. In 2018 and 2019, no wild birds positive for RT-PCR were detected even though 120 and 56 were tested, respectively. Absence of RT-PCR positivity does not necessarily mean lack of WNV circulation. In fact, in 2018, positivity for WNV by SNT was detected in an age-category 3 (i.e., born in 2018) Eurasian magpies (*Pica pica*) sampled in the same area where WNV had been reported in 2017 [34], which suggested that the virus might have overwintered in the area and recirculated in the following year. Furthermore, in 2018, a horse with symptoms compatible with WNV and IgM positive was detected in the municipality of Vilanova i la Geltrú, in central-eastern Catalonia [34]. In 2018, Europe experienced the largest WNV epidemic to date with 2083 human cases [35], which contrasts with the limited circulation observed in Catalonia in the same year. High WNV transmission in Europe in 2018, mainly in south-eastern areas, was associated with favourable climatic conditions (e.g., increased precipitation in March or increased temperatures in May) [17,21], which did not occur in Catalonia [36].

Phylogenetic analyses showed that the Spanish isolates from 2017 and 2020 belonged to the central-southern European WNV-L2 clade [30]. This clade also includes recent strains from Germany, France, the Czech Republic, Greece, and Italy, where important outbreaks in humans and animals have occurred [13,16,18,20,37,38,39]. Our results corroborate the spread of this cluster to southern and western areas of the European continent, and represent the westernmost point in Europe this WNV lineage has reached and established to date. Furthermore, these Spanish isolates were closely related with the Italian (2013) and the French (2018) ones, constituting a monophyletic group diverging from the Austrian-German group, with representatives from 2014 to 2020 [37].

A subsequent phylogenetic analysis revealed that the Spanish isolates specifically grouped to an Italian cluster, namely the Lombardy cluster [31]. This cluster, also known as clade B, has shown more ability to spread than the Veneto cluster (also known as clade A) [20]. Indeed, the Veneto cluster apparently became extinct in 2013–2014, as no new sequences from this clade (neither from Italy nor from other countries) have been detected since then and, remarkably, none were collected in Italy in 2018 during the largest epidemic of WNV infection [16,20]. Even more, the Lombardy cluster, which has been detected in the Veneto region since 2014 (in Verona, Vicenza, Rovigo, Venice, and Treviso regions), has even replaced the Veneto cluster, becoming the dominant one in the area [21]. Besides, the Lombardy cluster, which initially caused outbreaks in consecutive years in northern Italy (2013–2014) [31], was able to spread from northern Italian regions to north-eastern Spain in 2017 and south-eastern France in 2017 or 2018 (considering that the 2017 human cases and the WNV-L2 confirmed outbreaks in birds in 2018 reported in the same French area likely shared the same origin) [16]. This replacement of strains in Italy, followed by a successful spread towards Spain and France, suggests that this variant from the Lombardy cluster might have acquired improved fitness [21]. Moreover, this variant has demonstrated to have the ability to cause outbreaks at least in wild birds [16] and humans [21], and probably in horses [40]. Accordingly, the phylogenetic analyses suggest one single introduction of WNV-L2 from northern Italy into France and Spain, probably via infected birds, as has been usually hypothesised [16].

Our phylogenetic analyses together with the seropositivity found before and after the first detection of WNV-L2 [23,41] suggest the presence and overwintering of WNV in the region. Furthermore, it is unknown whether the WNV seropositivity in the region was due to WNV-L1 or WNV-L2. While only the latter has been identified in this territory, silent WNV-L1 circulation should not be discarded. Therefore, the molecular analysis of different samples is essential in order to characterise isolates and study the spread and evolution of WNV over time.

In the same way, it is important to point out the close phylogenetic relationship between certain Italian and Spanish isolates, as has been highlighted in this study of WNV-L2 and in previous ones focused on WNV-L1 [42]. This fact could reveal a particular dissemination route from Italy to Spain, although this hypothesis could be biased due to the absence of sequences from other western Mediterranean countries.

Likewise, while the isolate AC568 (Lleida, 2017) displayed phenotypic characteristics of isolates of high virulence in mouse models, according to previously described classification in relation to plaque size in cell culture [6,32,33], the isolate AC913 (Lleida, 2020) showed characteristics of moderate virulence (similar to B956 strain) [6]. On the other hand, isolates AC923 and AC924 (Tarragona, 2020) presented an intermediate phenotype between moderate and high virulence. This presumable virulence estimated in vitro might correlate with the virulence observed in the affected birds, although it is uncertain if it holds a link to the horse outbreaks occurred in the region during 2020 and 2021 seasons [40,43]. In any case, these plaque size assays must be considered only rough estimates to approach virulence of WNV strains, and further in vivo studies of pathogenicity in vertebrate hosts will be necessary in order to accurately evaluate the virulence of these new strains.

Wild raptors, especially northern goshawks, seem to be particularly susceptible to WNV neuro-invasive infections since they have been repeatedly found affected due to WNV infection in different countries in Europe [9,10,12,16,22,38,44,45,46]. In fact, from more than 350 birds sampled over 2017–2020, only five individuals were WNV positive by PCR, all of them northern goshawks that finally died or were euthanised due to the infection. This fact could be explained either because of their predation patterns, as they mainly feed on Passeriformes [46,47], which could be infected, and this may result in a subsequent infection of the predator [2], or due to mosquito feeding preferences [48].

Samples tested in this study were obtained from individuals of two WRC from Catalonia, highlighting the key role that these kind of centres can play in the surveillance and detection of emerging pathogens, as has been previously observed in other instances in Spain [41,49].

Finally, the detection of WNV-L2 in these northern goshawks in combination with the detection of specific antibodies against WNV in birds and horses from Catalonia [23] indicates an increased risk of WNV spillover into the human population in this area. Since WNV-L2 has been responsible for the largest outbreak of WNV in Europe in 2018 [35], it is necessary to reinforce WNV surveillance in Spain in order to better anticipate future outbreaks in humans.

## 5. Conclusions

This study confirmed the presence and overwintering of WNV-L2 in north-eastern Spain between 2017 and 2020. Northern goshawk was the affected species, further confirming the susceptibility of these raptors to WNV infection. In addition, this work highlighted the spread of WNV-L2 from central regions of Europe (such as Italy) to the west, causing outbreaks in France and Spain. Taking into account the results of this study and the situation observed in neighbouring countries (such as Italy, Germany, or Greece), the risk of a further spillover to humans is high. Thus, it is essential to reinforce surveillance in the country, under a One-Health approach, in order to provide an early warning and to take the appropriate prevention and control measures.

## Figures and Tables

**Table 1:** Analysis of the collected samples from the northern goshawks by WNV real-time RT-PCR [26].

Specimens	Sample Type/Tissue (Ct)				
Northern Goshawks (*Accipiter gentilis*)	Encephalon	Kidney	Liver	Heart	Lung
AC568	15.18	35.91	neg	neg	neg
AC913	14.50	28.73	34.02	31.34	31.26
AC923	14.47	25.99	29.90	30.21	29.02
AC924	18.11	20.85	30.17	27.71	27.88

**Table 2:** Comparison of the amino acid substitutions between the Spanish isolates and representatives of the Lombardy and Veneto clusters of WNV-L2. The isolate from Hungary 2004 was used as the reference sequence. Black dot indicates the same amino acid as the reference sequence. Amino acids that are unique in Spanish WNV-L2 polyproteins are highlighted in bold.

		Initial Reference	Veneto Cluster	Lombardy Cluster					
Viral Protein	Amino Acid Position	DQ116961 Hungary 2004 (Goshawk)	KF647249 Italy 2013 Rovigo (Human)	KF823806 Italy 2013 Mantova (Human)	MT863560 France 2018 (Common Buzzard)	AC568 Spain 2017 Lleida (Northern Goshawk)	AC913 Spain 2020 Lleida (Northern Goshawk)	AC923 Spain 2020 Tarragona (Northern Goshawk)	AC924 Spain 2020 Tarragona (Northern Goshawk)
C	34	M	·	·	·	·	·	**V**	·
prM	20	T	A	·	·	·	·	·	·
	29	A	·	·	·	·	·	·	**V**
E	88	S	P	P	P	P	P	P	P
	159	I	T	T	T	T	T	T	T
NS1	35	Y	·	H	H	H	H	H	H
	41	G	·	·	·	·	**S**	·	·
	69	G	E	E	E	E	E	E	E
	92	K	·	·	E	·	·	·	·
	113	M	·	·	·	·	·	**I**	·
	123	I	·	·	·	**F**	**F**	**F**	**F**
	146	A	V	·	·	·	·	·	·
	293	H	·	·	·	**R**	**R**	**R**	**R**
NS2A	1	H	Y	Y	Y	Y	Y	Y	Y
	192	S	C	C	C	C	C	C	C
NS4B	11	N	·	·	·	·	·	**S**	**S**
NS5	26	A	T	T	T	T	T	T	T
	203	Y	H	·	·	·	·	·	·
	277	T	·	·	·	**I**	**I**	**I**	**I**
	279	K	·	R	·	·	·	·	·
	299	A	T	T	T	T	T	T	T
	340	N	S	·	·	·	·	·	·
	599	R	·	·	·	·	·	·	**I**
	886	V	A	·	·	·	·	·	·

## Data Availability

The sequences presented in this study are openly available in GenBank database.

## Supplementary Materials

The following supporting information can be downloaded at: https://www.mdpi.com/article/10.3390/v14030569/s1, Table S1: Summary of the wild bird species analysed during the passive surveillance program carried out between 2017 and 2020; Table S2: Primers’ sequences used in this study for the whole genome sequencing of the first Spanish WNV-L2 isolates; Table S3: Percent identity matrix of the polyprotein between the Spanish isolates and representatives of the Lombardy and Veneto clusters of WNV-L2. The isolate from Hungary 2004 was used as the reference sequence.

## References

[1] Mencattelli G., Ndione M.H.D., Rosà R., Marini G., Diagne C.T., Diagne M.M., Fall G., Faye O., Diallo M., Faye O. (2022). Epidemiology of West Nile Virus in Africa: An Underestimated Threat. PLoS Negl. Trop. Dis..

[2] Komar N., Langevin S., Hinten S., Nemeth N., Edwards E., Hettler D., Davis B., Bowen R., Bunning M. (2003). Experimental Infection of North American Birds with the New York 1999 Strain of West Nile Virus. Emerg. Infect. Dis..

[3] García San Miguel Rodríguez-Alarcón L., Fernández-Martínez B., Sierra Moros M.J., Vázquez A., Julián Pachés P., García Villacieros E., Gómez Martín M.B., Figuerola Borras J., Lorusso N., Ramos Aceitero J.M. (2021). Unprecedented Increase of West Nile Virus Neuroinvasive Disease, Spain, Summer 2020. Eurosurveillance.

[4] Rizzoli A., Jimenez-Clavero M.A., Barzon L., Cordioli P., Figuerola J., Koraka P., Martina B., Moreno A., Nowotny N., Pardigon N. (2015). The Challenge of West Nile Virus in Europe : Knowledge Gaps and Research Priorities. Eurosurveillance.

[5] Habarugira G., Suen W.W., Hobson-Peters J., Hall R.A., Bielefeldt-Ohmann H. (2020). West Nile Virus: An Update on Pathobiology, Epidemiology, Diagnostics, Control and “One Health” Implications. Pathogens.

[6] Pérez-Ramírez E., Llorente F., Del Amo J., Fall G., Sall A.A., Lubisi A., Lecollinet S., Vázquez A., Jiménez-Clavero M.Á. (2017). Pathogenicity Evaluation of Twelve West Nile Virus Strains Belonging to Four Lineages from Five Continents in a Mouse Model: Discrimination between Three Pathogenicity Categories. J. Gen. Virol..

[7] Murgue B., Murri S., Triki H., Deubel V., Zeller H.G. (2006). West Nile in the Mediterranean Basin: 1950–2000. Ann. N. Y. Acad. Sci..

[8] Barrett A.D.T. (2018). West Nile in Europe: An Increasing Public Health Problem. J. Travel Med..

[9] Bakonyi T., Ivanics É., Erdélyi K., Ursu K., Ferenczi E., Weissenböck H., Nowotny N. (2006). Lineage 1 and 2 Strains of Encephalitic West Nile Virus, Central Europe. Emerg. Infect. Dis..

[10] Bakonyi T., Ferenczi E., Erdélyi K., Kutasi O., Csörgo T., Seidel B., Weissenböck H., Brugger K., Bán E., Nowotny N. (2013). Explosive Spread of a Neuroinvasive Lineage 2 West Nile Virus in Central Europe, 2008/2009. Vet. Microbiol..

[11] Papa A., Bakonyi T., Xanthopoulou K., Vázquez A., Tenorio A., Nowotny N. (2011). Genetic Characterization of West Nile Virus Lineage 2, Greece, 2010. Emerg. Infect. Dis..

[12] Petrović T., Blázquez A.B., Lupulović D., Lazić G., Escribano-Romero E., Fabijan D., Kapetanov M., Lazić S., Saiz J.C. (2013). Monitoring West Nile Virus (WNV) Infection in Wild Birds in Serbia during 2012: First Isolation and Characterisation of WNV Strains from Serbia. Eurosurveillance.

[13] Barzon L., Pacenti M., Franchin E., Lavezzo E., Masi G., Squarzon L., Pagni S., Toppo S., Russo F., Cattai M. (2013). Whole Genome Sequencing and Phylogenetic Analysis of West Nile Virus Lineage 1 and Lineage 2 from Human Cases of Infection, Italy, August 2013. Eurosurveillance.

[14] Platonov A.E., Karan L.S., Shopenskaia T.A., Fedorova M.V., Koliasnikova N.M., Rusakova N.M., Shishkina L.V., Arshba T.E., Zhuravlev V.I., Govorukhina M.V. (2011). Genotyping of West Nile Fever Virus Strains Circulating in Southern Russia as an Epidemiological Investigation Method: Principles and Results. Zhurnal Mikrobiol. Epidemiol. Immunobiol..

[15] Sirbu A., Ceianu C.S., Panculescu-Gatej R.I., Vázquez A., Tenorio A., Rebreanu R., Niedrig M., Nicolescu G., Pistol A. (2011). Outbreak of West Nile Virus Infection in Humans, Romania, July to October 2010. Eurosurveillance.

[16] Beck C., Goffart I.L., Franke F., Gonzalez G., Dumarest M., Lowenski S., Blanchard Y., Lucas P., de Lamballerie X., Grard G. (2020). Contrasted Epidemiological Patterns of West Nile Virus Lineages 1 and 2 Infections in France from 2015 to 2019. Pathogens.

[17] Ziegler U., Lühken R., Keller M., Cadar D., Van Der Grinten E., Michel F., Albrecht K., Eiden M., Rinder M., Lachmann L. (2019). West Nile Virus Epizootic in Germany, 2018. Antivir. Res..

[18] Zelená H., Kleinerová J., Šikutová S., Straková P., Kocourková H., Stebel R., Husa P., Husa P., Tesařová E., Lejdarová H. (2021). First Autochthonous West Nile Lineage 2 and Usutu Virus Infections in Humans, July to October 2018, Czech Republic. Pathogens.

[19] Vlaskamp D.R.M., Thijsen S.F.T., Reimerink J., Hilkens P., Bouvy W.H., Bantjes S.E., Vlaminckx B.J.M., Zaaijer H., van den Kerkhof H.H.T.C., Raven S.F.H. (2020). First Autochthonous Human West Nile Virus Infections in the Netherlands, July to August 2020. Eurosurveillance.

[20] Veo C., Della Ventura C., Moreno A., Rovida F., Percivalle E., Canziani S., Torri D., Calzolari M., Baldanti F., Galli M. (2019). Evolutionary Dynamics of the Lineage 2 West Nile Virus That Caused the Largest European Epidemic: Italy 2011–2018. Viruses.

[21] Pacenti M., Sinigaglia A., Franchin E., Pagni S., Lavezzo E., Montarsi F., Capelli G., Barzon L. (2020). Human West Nile Virus Lineage 2 Infection: Epidemiological, Clinical, and Virological Findings. Viruses.

[22] Aguilera-Sepúlveda P., Gómez-Martín B., Agüero M., Jiménez-Clavero M.Á., Fernández-Pinero J. (2021). A New Cluster of West Nile Virus Lineage 1 Isolated from a Northern Goshawk in Spain. Transbound. Emerg. Dis..

[23] Napp S., Llorente F., Beck C., Jose-Cunilleras E., Soler M., Pailler-García L., Amaral R., Aguilera-Sepúlveda P., Pifarér M., Molina-López R. (2021). Widespread Circulation of Flaviviruses in Horses and Birds in Northeastern Spain (Catalonia) between 2010 and 2019. Viruses.

[24] Busquets N., Laranjo-González M., Soler M., Nicolás O., Rivas R., Talavera S., Villalba R., San Miguel E., Torner N., Aranda C. (2019). Detection of West Nile Virus Lineage 2 in North-Eastern Spain (Catalonia). Transbound. Emerg. Dis..

[25] Petrovic T., Šekler M., Petric D., Vidanovic D., Debeljak Z., Lazic G., Lupulovic D., Kavran M., Samojlovic M., Ignjatovic Cupina A. (2021). Intensive West Nile Virus Circulation in Serbia in 2018 Results of Integrated Surveillance Program. Pathogens.

[26] Linke S., Ellerbrok H., Niedrig M., Nitsche A., Pauli G. (2007). Detection of West Nile Virus Lineages 1 and 2 by Real-Time PCR. J. Virol. Methods.

[27] Scaramozzino N., Crance J., Jouan A., De Briel D.A., Stoll F., Garin D. (2001). Comparison of Flavivirus Universal Primer Pairs and Development of a Rapid, Highly Sensitive Heminested Reverse Transcription-PCR Assay for Detection of Flaviviruses Targeted to a Conserved Region of the NS5 Gene Sequences. J. Clin. Microbiol..

[28] Jourdain E. (2006). Oiseaux Sauvages et Virus West Nile: Étude Éco-Épidémiologique en Camargue. Ph.D. Thesis.

[29] Payne A.F., Binduga-Gajewska I., Kauffman E.B., Kramer L.D. (2006). Quantitation of Flaviviruses by Fluorescent Focus Assay. J. Virol. Methods.

[30] Ravagnan S., Montarsi F., Cazzin S., Porcellato E., Russo F., Palei M., Monne I., Savini G., Marangon S., Barzon L. (2015). First Report Outside Eastern Europe of West Nile Virus Lineage 2 Related to the Volgograd 2007 Strain, Northeastern Italy, 2014. Parasites Vectors.

[31] Barzon L., Papa A., Lavezzo E., Franchin E., Pacenti M., Sinigaglia A., Masi G., Trevisan M., Squarzon L., Toppo S. (2015). Phylogenetic Characterization of Central/Southern European Lineage 2 West Nile Virus: Analysis of Human Outbreaks in Italy and Greece, 2013–2014. Clin. Microbiol. Infect..

[32] Davis C.T., Beasley D.W.C., Guzman H., Siirin M., Parsons R.E., Tesh R.B., Barrett A.D.T. (2004). Emergence of Attenuated West Nile Virus Variants in Texas, 2003. Virology.

[33] Jia Y., Moudy R.M., Dupuis A.P., Ngo K.A., Maffei J.G., Jerzak G.V.S., Franke M.A., Kauffman E.B., Kramer L.D. (2007). Characterization of a Small Plaque Variant of West Nile Virus Isolated in New York in 2000. Virology.

[34] Napp S., Montalvo T., Piñol-Baena C., Gómez-Martín M.B., Nicolás-Francisco O., Soler M., Busquets N. (2019). Usefulness of Eurasian Magpies (*Pica pica*) for West Nile Virus Surveillance in Non-Endemic and Endemic Situations. Viruses.

[35] European Centre for Disease Prevention and Control (2018). Epidemiological Update: West Nile Virus Transmission Season in Europe. https://www.ecdc.europa.eu/en/news-events/epidemiological-update-west-nile-virus-transmission-season-europe-2018.

[36] Copernicus Climate Change Service Monthly Summaries of Precipitation, Relative Humidity and Soil Moisture 2018. https://climate.copernicus.eu/.

[37] Ziegler U., Santos P.D., Groschup M.H., Hattendorf C., Eiden M., Höper D., Eisermann P., Keller M., Michel F., Klopfleisch R. (2020). West Nile Virus Epidemic in Germany Triggered by Epizootic Emergence, 2019. Viruses.

[38] Hubálek Z., Tomešek M., Kosina M., Šikutová S., Straková P., Rudolf I. (2019). West Nile Virus Outbreak in Captive and Wild Raptors, Czech Republic, 2018. Zoonoses Public Health.

[39] Papa A., Papadopoulou E., Chatzixanthouliou C., Glouftsios P., Pappa S., Pervanidou D., Georgiou L. (2019). Emergence of West Nile Virus Lineage 2 Belonging to the Eastern European Subclade, Greece. Arch. Virol..

[40] Ministerio de Agricultura, Pesca y Alimentación Consulta de Notificación de Enfermedades de los Animales de Declaración Obligatoria. https://servicio.mapa.gob.es/rasve/Publico/Publico/BuscadorFocos.aspx.

[41] Alba A., Allepuz A., Napp S., Soler M., Selga I., Aranda C., Casal J., Pages N., Hayes E.B., Busquets N. (2014). Ecological Surveillance for West Nile in Catalonia (Spain), Learning from a Five-Year Period of Follow-Up. Zoonoses Public Health.

[42] Sotelo E., Fernández-Pinero J., Llorente F., Vázquez A., Moreno A., Agüero M., Cordioli P., Tenorio A., Jiménez-Clavero M.Á. (2011). Phylogenetic Relationships of Western Mediterranean West Nile Virus Strains (1996–2010) Using Full-Length Genome Sequences: Single or Multiple Introductions?. J. Gen. Virol..

[43] European Centre for Disease Prevention and Control (ECDC) Weekly Updates: 2021 West Nile Virus Transmission Season. https://www.ecdc.europa.eu/en/west-nile-fever/surveillance-and-disease-data/disease-data-ecdc.

[44] Feyer S., Bartenschlager F., Bertram C.A., Ziegler U., Fast C., Klopfleisch R., Müller K. (2021). Clinical, Pathological and Virological Aspects of Fatal West Nile Virus Infections in Ten Free-Ranging Goshawks (*Accipiter gentilis*) in Germany. Transbound. Emerg. Dis..

[45] Hubálek Z., Kosina M., Rudolf I., Mendel J., Straková P., Tomešek M. (2018). Mortality of Goshawks (*Accipiter gentilis*) Due to West Nile Virus Lineage 2. Vector-Borne Zoonotic Dis..

[46] Vidaña B., Busquets N., Napp S., Perez-Ramírez E., Jiménez-Clavero M.Á., Johnson N. (2020). The Role of Birds of Prey in West Nile Virus Epidemiology. Vaccines.

[47] García-Salgado G., Rebollo S., Pérez-Camacho L., Martínez-Hesterkamp S., Navarro A., Fernández-Pereira J.M. (2015). Evaluation of Trail-Cameras for Analyzing the Diet of Nesting Raptors Using the Northern Goshawk as a Model. PLoS ONE.

[48] Victoriano Llopis I., Tomassone L., Grego E., Serrano E., Mosca A., Vaschetti G., Andrade D., Rossi L. (2016). Evaluating the Feeding Preferences of West Nile Virus Mosquito Vectors Using Bird-Baited Traps. Parasites Vectors.

[49] Bravo-Barriga D., Aguilera-Sepúlveda P., Guerrero-Carvajal F., Llorente F., Reina D., Pérez-Martín J.E., Jiménez-Clavero M.Á., Frontera E. (2021). West Nile and Usutu Virus Infections in Wild Birds Admitted to Rehabilitation Centres in Extremadura, Western Spain, 2017–2019. Vet. Microbiol..

