# Supplementary material for: West Nile Virus Lineage 2 Spreads Westwards in Europe and Overwinters in North-Eastern Spain (2017–2020)
Source: Viruses. 2022 Mar 9;14(3):569. doi: 10.3390/v14030569 (PMC8951896; doi:10.3390/v14030569)
Supplement: Supplementary file 1 [file viruses-14-00569-s001.zip › Table S1.pdf]

Table S1. Summary of the wild bird species analysed during the passive surveillance program carried out between 2017 and 2020.

N: number of individuals sampled.

**Family**	**Specie**	**N**
Accipitridae	*Accipiter gentilis*	10
	Aegypius monachus	1
	Buteo buteo	1
	Circus aeruginosus	2
	Gypaetus barbatus	2
	Milvus milvus	1
	Pernis apivorus	1
Anatidae	*Alopochen aegyptiaca*	1
	Anas platyrhynchos	50
	Anser anser	4
	Aythya ferina	1
	Cairina moschata	4
	Cygnus olor	2
	Spatula clypeata	1
	Tadorna tadorna	2
Ardeidae	*Ardea cinerea*	2
	Bubulcus ibis	15
	Egretta garzetta	2
Charadriidae	*Vanellus vanellus*	1
Ciconiidae	*Ciconia ciconia*	24
Columbidae	*Columba livia*	36
	Columba palumbus	6
	Streptopelia decaocto	51
Corvidae	*Corvus corax*	1
	Corvus monedula	1
	Garrulus glandarius	1
	Pica pica	84
Falconidae	*Falco tinnunculus*	3
Gruidae	*Grus grus*	1
Laniidae	*Lanius minor*	4
Laridae	*Larus melanocephalus*	1
	Larus michahellis	3
	Larus ridibundus	4
Paridae	*Cyanistes caeruleus*	1
	Parus major	6
Passeridae	*Passer domesticus*	9
	Passer montanus	2
Phalacrocoracidae	*Phalacrocorax carbo*	4
Phasianidae	*Gallus gallus*	4
Phoenicopteridae	*Phoenicopterus roseus*	1

Phylloscopidae	*Phylloscopus collybita*	1
Picidae	*Picus viridis*	1
Psittacidae	*Myiopsitta monachus*	1
Rallidae	*Fulica atra*	1
	Gallinula chloropus	1
Strigidae	*Asio otus*	1
	Athene noctua	2
	Otus scops	1
	Strix aluco	1
Sturnidae	*Sturnus unicolor*	1
	Sturnus vulgaris	9
Turdidae	*Turdus merula*	1
	Turdus philomelos	2
**Total**		**373**
