# Supplementary material for: West Nile Virus Lineage 2 Spreads Westwards in Europe and Overwinters in North-Eastern Spain (2017–2020)
Source: Viruses. 2022 Mar 9;14(3):569. doi: 10.3390/v14030569 (PMC8951896; doi:10.3390/v14030569)
Supplement: Supplementary file 1 [file viruses-14-00569-s001.zip › Table S2.pdf]

Table S2. Primers' sequences used in this study for the whole genome sequencing of the first Spanish WNV-L2 isolates. + symbol indicates LNA residues.

Primer pairs	Primer ID* (* ref. KF647251)	Sequence	Author (* personal communications)
1	WN_5'_end	AGTAGTTCGCCTGTGTGAGCTGAC	Jourdain et al., 2006
	WNL2_457c	GTCACAGCTCCAGCACAAGCGAT	M. Elizalde and J. Fernández-Pinero *
2	WNL2_310	CAGCGATGAAGCATCTCTTGAGTT	M. Elizalde and J. Fernández-Pinero *
	WNL2_941c	TGCCACCAGGAGCAATAGGA	L. Barzon *
3	WNL2_857	CCTCGTTGCAGCGGTCATGG	M. Elizalde and J. Fernández-Pinero *
	WNL2_1526c	ACAGTCAACCGTGACCTCACCAT	M. Elizalde and J. Fernández-Pinero *
4	WNL2_1390	CGACGACTGTTGAATCTCATGGC	M. Elizalde and J. Fernández-Pinero *
	WNL2_2034c	ATGGATTCACGGTCACCAGTCTTC	M. Elizalde and J. Fernández-Pinero *
5	WNL2_1904	CACTGGCCATGGAACGGT	M. Elizalde and J. Fernández-Pinero *
	WNL2_2492c	CTCTTGCCTGCCAATATCAATG	M. Elizalde and J. Fernández-Pinero *
6	WNL2_2308	CACTCTTTGGAGGGATGTCCTG	M. Elizalde and J. Fernández-Pinero *
	WNL2_2962c	AGGAACATGCGAGTGCTTGTC	M. Elizalde and J. Fernández-Pinero *
7	WNL2_2829	GCTCCAGAACTAGCTAACAACACCTT	M. Elizalde and J. Fernández-Pinero *
	WNL2_3457c	TCCATTCCATACCAACAGCCA	M. Elizalde and J. Fernández-Pinero *
8	WNL2_3317	AAGTGATAGTTGCGGACACCGT	M. Elizalde and J. Fernández-Pinero *
	WNL2_3942c	TGAGAATCATCCACGCGACG	L. Barzon *
9	WNL2_3860	GGCTTACTATGACGCCAAGA	L. Barzon *
	WNL2_4453c	CCATCATCATCCAGCCTAAC	L. Barzon *
10	WNL2_4356	GATATGTGGATCGAGAGGACGG	Jourdain et al., 2006
	WNL2_4995c	CAGTGGGATAGTCTAGCGTAACTG	M. Elizalde and J. Fernández-Pinero *
11	WNL2_4877	TGAGGTCCAAATGATTGTCGTG	M. Elizalde and J. Fernández-Pinero *
	WNL2_5604c	TGTCCGAGATAGGAGCATTG	L. Barzon *
12	WNL2_5447	AATGGATGAAGCCCATTTCACG	M. Elizalde and J. Fernández-Pinero *
	WN5808c	GACAAAGTCCCAATCATCGTTCTT	Jourdain et al., 2006
13	WNL2_5629	GCCTGGAACACTGGATATGA	L. Barzon *
	WNL2_6244c	TAAGCGAGCCAGACTGGTAA	L. Barzon *
14	WNL2_6126	TATCAGCCTGAGCGCGAGAA	L. Barzon *
	WNL2_6593c	CTCCTCAAGAGCCATCCTGTGA	M. Elizalde and J. Fernández-Pinero *
15	WNL2_6469	TCGTTGAGGTGCTCGGGAGA	M. Elizalde and J. Fernández-Pinero *
	WNL2_7057c	ACGGCTGTCGTTACGGCATA	M. Elizalde and J. Fernández-Pinero *
16	WN6895	AATGAGATGGGTTGGCTGGACAAGA	Jourdain et al., 2006
	WNL2_7609c	AGCCATCCTCCTCGCATGATGT	M. Elizalde and J. Fernández-Pinero *
17	WNL2_7492	GAGAGGCTGGAATTCTGACT	L. Barzon *
	WNL2_8145c	GTTCTTCTACCTCGGCACTT	L. Barzon *
18	WNL2_8077	CATCAGAAGCGAGCGACACA	L. Barzon *
	WNL2_8603c	ATAGCTTCCGTGGTAGTTCCAG	M. Elizalde and J. Fernández-Pinero *
19	WNL2_8492	CTGATACTAGCAAGATCAAGAACCGA	M. Elizalde and J. Fernández-Pinero *
	WNL2_8993c	ACGCTCCTCATCCACCATCT	M. Elizalde and J. Fernández-Pinero *
20	WNL2_8901	CTAGGAGCGATGTTTGAAGAACAGA	M. Elizalde and J. Fernández-Pinero *
	WNL2_9464c	TCTGGAGATGACATCCATCACAGT	M. Elizalde and J. Fernández-Pinero *
21	WNL2_9367	TAGCGCGGTCCATCATTGAG	L. Barzon *
	WNL2_10020c	TGGCGTTAGCCATCAATCTCA	M. Elizalde and J. Fernández-Pinero *
22	WNL2_9955	CATACGCGCAGATGTGGCT	M. Elizalde and J. Fernández-Pinero *
	WNL2_10616c	CGTGGATCACCTCGCAACTT	M. Elizalde and J. Fernández-Pinero *
23	JE_10478	GTACA+CGGTGCTGY+CTG	M. Elizalde and J. Fernández-Pinero *
	WNL2_11011c	CACCTATATCGGCGCACTGT	M. Elizalde and J. Fernández-Pinero *
