# Supplementary material for: West Nile Virus Lineage 2 Spreads Westwards in Europe and Overwinters in North-Eastern Spain (2017–2020)
Source: Viruses. 2022 Mar 9;14(3):569. doi: 10.3390/v14030569 (PMC8951896; doi:10.3390/v14030569)
Supplement: Supplementary file 1 [file viruses-14-00569-s001.zip › Table S3.pdf]

Table S3. Percent identity matrix of the polyprotein between the Spanish isolates and representatives of the Lombardy and Veneto clusters of WNV-L2. The isolate from Hungary 2004 was used as the reference sequence.

	**DQ116961 Hungary 2004**	**KF647249 Italy 2013 Rovigo**	**MT863560 France 2018**	**KF823806 Italy 2013 Mantova**	**AC923 Spain 2020 Tarragona**	**AC924 Spain 2020 Tarragona**	**AC913 Spain 2020 Lleida**	**AC568 Spain 2017 Lleida**
**DQ116961 Hungary 2004**	**100.00**	99.65	99.74	99.74	99.59	99.59	99.65	99.68
**KF647249 Italy 2013 Rovigo**	99.65	**100.00**	99.80	99.80	99.65	99.65	99.71	99.74
**MT863560 France 2018**	99.74	99.80	**100.00**	99.94	99.80	99.80	99.85	99.88
**KF823806 Italy 2013 Mantova**	99.74	99.80	99.94	**100.00**	99.80	99.80	99.85	99.88
**AC923 Spain 2020 Tarragona**	99.59	99.65	99.80	99.80	**100.00**	99.88	99.88	99.91
**AC924 Spain 2020 Tarragona**	99.59	99.65	99.80	99.80	99.88	**100.00**	99.88	99.91
**AC913 Spain 2020 Lleida**	99.65	99.71	99.85	99.85	99.88	99.88	**100.00**	99.97
**AC568 Spain 2017 Lleida**	99.68	99.74	99.88	99.88	99.91	99.91	99.97	**100.00**
